# The role of *Staphylococcus aureus* quorum sensing in cutaneous and systemic infections

**DOI:** 10.1186/s41232-024-00323-8

**Published:** 2024-03-01

**Authors:** Yuriko Yamazaki, Tomoka Ito, Masakazu Tamai, Seitaro Nakagawa, Yuumi Nakamura

**Affiliations:** 1 Cutaneous Allergy and Host Defense, Immunology Frontier Research Center, Osaka, University, Osaka, 565-0871 Japan; 2 Department of Dermatology, Osaka University Graduate School of Medicine, Osaka, 565-0871 Japan

**Keywords:** *Staphylococcus aureus*, Accessory gene regulator, Quorum sensing, Infectious diseases, Skin infection, Atopic dermatitis, Systemic infection

## Abstract

**Background:**

*Staphylococcus aureus* is a leading cause of human bacterial infections worldwide. It is the most common causative agent of skin and soft tissue infections, and can also cause various other infections, including pneumonia, osteomyelitis, as well as life-threatening infections, such as sepsis and infective endocarditis. The pathogen can also asymptomatically colonize human skin, nasal cavity, and the intestine. *S. aureus* colonizes approximately 20–30% of human nostrils, being an opportunistic pathogen for subsequent infection. Its strong ability to silently spread via human contact makes it difficult to eradicate *S*. *aureus*. A major concern with *S*. *aureus* is its capacity to develop antibiotic resistance and adapt to diverse environmental conditions. The variability in the accessory gene regulator (Agr) region of the genome contributes to a spectrum of phenotypes within the bacterial population, enhancing the likelihood of survival in different environments. Agr functions as a central quorum sensing (QS) system in *S*. *aureus*, allowing bacteria to adjust gene expression in response to population density. Depending on Agr expression, *S*. *aureus* secretes various toxins, contributing to virulence in infectious diseases. Paradoxically, expressing Agr may be disadvantageous in certain situations, such as in hospitals, causing *S*. *aureus* to generate Agr mutants responsible for infections in healthcare settings.

**Main body:**

This review aims to demonstrate the molecular mechanisms governing the diverse phenotypes of *S*. *aureus*, ranging from a harmless colonizer to an organism capable of infecting various human organs. Emphasis will be placed on QS and its role in orchestrating *S*. *aureus* behavior across different contexts.

**Short conclusion:**

The pathophysiology of *S*. *aureus* infection is substantially influenced by phenotypic changes resulting from factors beyond Agr. Future studies are expected to give the comprehensive understanding of *S. aureus* overall profile in various settings.

## Background

### *S. aureus* resistance and adaptation to the ecological niche

Throughout human history, we have consistently battled bacteria. However, bacteria have persistently sought out vulnerabilities in our attempt and adapted to their ever-changing environment. The mortality rate from systemic *S*. *aureus* infections was approximately 80% before the discovery of antibiotics [1]. The discovery of penicillin in 1928 [2] and its clinical use temporarily decreased the death toll due to bacterial pneumonia and meningitis during World War II. However, only 2 years after the clinical introduction of penicillin, the penicillin-resistant *S*. *aureus* strains developed [1] and became predominant worldwide: 80% of clinical isolates were resistant to penicillin by 1945 and penicillin-resistant *S*. *aureus* became a pandemic throughout the late 1950s and early 1960s [1]. These strains encode β-lactamase, which is capable of hydrolyzing the β-lactam ring of penicillin [1]. To overcome this, methicillin, the semisynthetic β-lactamase-resistant antibiotic, was developed in 1959, although it led to the emergence of methicillin-resistant *S*. *aureus* (MRSA) soon after in 1961 [3]. MRSA arises due to the presence of the* mecA* gene. This gene encodes a modified penicillin-binding protein with reduced affinity for methicillin and other β-lactam antibiotics [3]. Importantly, *mecA* is situated within the Staphylococcal cassette chromosome mec (SCC*mec*), a mobile genetic element (MGE) that has the capability to transfer between bacterial strains [3]. This transferability of SCCmec contributes to the spread of methicillin resistance among *S*. *aureus* strains. MRSA spread worldwide over the next several decades and started to cause an endemic in hospitals and healthcare facilities, affecting immune-compromised hosts and causing life-threatening infections. Starting from the 1980s, MRSA spread globally to such an extent that many countries now report MRSA rates of 50% or higher among infective *S*. *aureus* isolates in hospitals [4]. For a while, MRSA was confined to only hospitals and healthcare settings. However, since early 1990s, MRSA started to cause outbreaks among otherwise healthy individual outside of the hospital settings, such as in sports teams, army recruits, or prisoners [5]. These novel MRSA strains, capable of infecting healthy individuals within the community, have been designated as community-associated (CA)-MRSA strains. In contrast, the traditional strains prevalent in hospital settings are referred to as hospital-acquired (HA)-MRSA strains. CA-MRSA infections are now prevalent and widespread worldwide [4]. Notably, CA-MRSA strains are more virulent and transmissible than are traditional HA-MRSA strains [6]. CA-MRSA gains methicillin resistance via small size SCC*mec*, type IV and V, which is attributed to less metabolic burdens of protein synthesis during replication, whereas HA-MRSA carries other large SCC*mec* types [7, 8]. It became clear that HA-MRSA and CA-MRSA differ in host selectivity and virulence. As the history proves, a formidable ability to adapt to a specific ecological niche, with the host immune system and environment, seems to be the core characteristics of *S. aureus* survival strategy. Currently, *S*. *aureus* is gaining new resistance to different antibiotics [9]. Multi-resistant *S*.* aureus* in hospitals not only leads to death and disability of immunocompromised hosts, but also prolongs illness of those who survive and requires more expensive medication, posing a financial challenge [10]. Understanding the fundamental bacterial property that enables MRSA to adapt to various environments and eventually gain resistance is an urgent need to fight against this bacterium. Recent advances in genome sequencing enabled us to understand bacterial genomic transition in detail [11]. In particular, the variation in the accessory gene regulator (Agr) region on the genome seems to generate a kaleidoscopic of phenotypes within the bacterial population and increases the likelihood of bacterial survival in versatile environments [12–14]. In this review, we will discuss how Agr regulates the bacterial phenotype in various infectious diseases.

### Agr quorum sensing in *S. aureus*

**Fig. 1:**
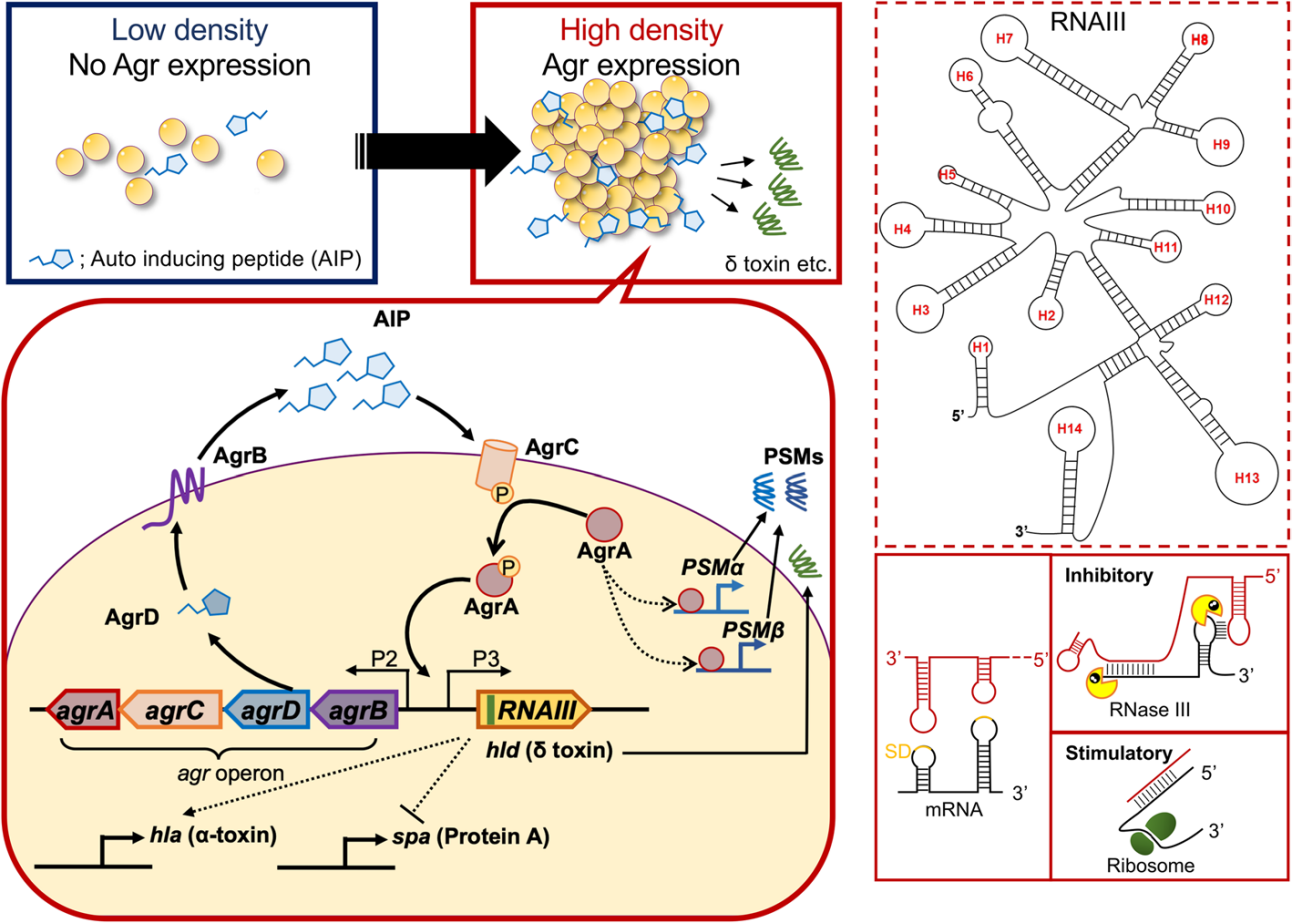
Agr quorum sensing in *Staphylococcus aureus*. *S*. *aureus* consistently releases AIP. When the population density and corresponding AIP reaches the threshold, the receptor on the cell surface, AgrC, is activated via autophosphorylation of the histidine kinase. The phosphate is transferred to AgrA, which in turn binds and activates two bidirectional promoters, P2 and P3 in *agr* operon. The P2 promoter drives the autoregulation circuit of Agr, by inducing the expression of agrBDCA operon, that encodes the machinery of the QS system. AgrD is cleaved by AgrB, a trans-membrane peptidase, to form of AIP. The P3 promoter regulates various toxins via RNAIII, which is a large regulatory RNA. RNAIII exhibits a complex secondary structure with several C-rich hairpin loops, many of which align with the Shine-Dalgarno sequence of targeted genes. These interactions can manifest as either inhibitory or stimulatory. RNAIII also encodes *hld*, a gene responsible for δ-toxin. *Agr* accessory gene receptor, *AIP* auto-inducing peptide, *QS* quorum-sensing, *SD* Shine-Dalgarno sequence

The quorum sensing (QS) system is the ability of bacteria to adjust gene expressions in response to their population density [15]. Many bacteria secrete chemical signaling molecules, called autoinducers, which vary between species [15]. When a bacterial population increases and the corresponding autoinducers reaches a threshold concentration, the signal activates a regulator that can induce or repress target genes [15]. Among the many traits controlled by QS is the expression of virulence factors, conjugation, biofilm formation [15]. *S*. *aureus* possesses an auto-regulatory operon, Agr system, as a QS function (Fig. 1) [16]. *S*. *aureus* consistently releases an exocrine auto-inducing peptide (AIP). AgrD is the 45–47 residue peptide precursor of AIP [16], which is proteolytically processed by AgrB, a trans-membrane peptidase. AgrB-mediated cleavage of AgrD results in the formation of AIP [17–19], which is transported to the extracellular space [20]. This peptide is typically seven to nine amino acids in length and features a distinctive five-residue thiolactone ring formed between the C-terminal end and a conserved cysteine residue [16]. When AIP reaches the threshold, the transmembrane receptor on the cell surface, AgrC, is activated via autophosphorylation of its histidine protein kinase (HPK) domain [16]. The phosphate of HPK is transferred to AgrA, which in turn binds and activates two bidirectional promoters, P2 and P3 in *agr* operon [16]. The P2 promoter drives the autoregulation circuit of Agr, by inducing the expression of the *agrBDCA* operon, that encodes the machinery of the QS system. In contrast, the P3 promoter regulates various toxins via RNAIII, a large regulatory RNA which has a complex secondary structure with several C-rich hairpin loops to interact with its target mRNAs [16, 21]. RNAIII also encodes the delta-hemolysin gene (*hld*), known as δ-toxin [16]. In the following section, we will discuss various toxins regulated by the Agr system. Their pathological role in infections will be discussed in subsequent sections.

### SarA, the upstream regulator of Agr

In addition to AgrA, other regulators are known to control *agr* expression [22]. Among these are SarA and SarR, both winged-helix DNA-binding proteins. SarA binds to the conserved regions, Sar boxes, within the promoter region of targeted genes [23, 24]. In association with Agr, SarA binds to the P2-P3 intergenic region in *agr* operon, induces DNA bending, and thus allow interaction of two AgrA dimers to result in the efficient recruitment of RNA polymerase and augmentation of RNAII transcription [25]. Without effective SarA enhancement, Agr operon can be only weakly activated [25]. Additionally, SarA binds to promoter regions of α-toxin and fibronectin-binding protein A to enhance their expressions [23, 24]. Therefore, SarA controls regulation of certain Agr-regulated virulence factors both directly and indirectly [26]. Contrary to SarA, SarR functions as a brake to attenuate Agr expression during the stationary phase, when cell growth reaches a confluence. During the post-exponential phase, SarR accumulates and binds to the *agr* promoter at a site that overlaps with SarA. This binding results in the displacement of SarA and the reversal of DNA bending [25]. Although not directly affected, *agr* P3 promoter is indirectly affected by the SarA/R system via its regulation on the P2 promoter and resulting *agr* operon machinery [25].

The SarA protein family is a collection of DNA-binding proteins homologous to SarA (SarR, SarS, SarT, SarU, SarV, SarX, SarZ, MgrA, and Rot) [22]. While each protein acts on various gene expressions independently, the SarA family also interact each other in a complexed manner and create a hierarchical regulatory cascade, affecting Agr expression in a complicated way. The interplay of the SarA family and its effect on Agr is only partially understood [20, 22].

### Phenol-soluble modulin

Phenol-soluble modulins (PSMs) are a family of small (2–5 kDa), amphipathic, α-helical peptides, including PSMα, PSMβ, and PSMγ (also called δ-toxin and delta-hemolysin) [27, 28] (Table 1). In addition to activating P2 and P3 promoter in the *agr* region, AgrA is capable of directly binding to the promoters of the PSMα (encoding PSMα1-α4) and PSMβ (encoding PSMβ1 and β2) [29]. The gene locus of δ-toxin (*hld*) is located in RNAIII region of *agr* operon thus transcribed by P3 promoter [30]. The essential virulence of PSM peptides rely on its cytolytic property, although not all PSMs from *S*. *aureus* are cytolytic. PSMα, especially PSMα3, has a pronounced ability to lyse human leukocytes, erythrocytes, and epithelial cells; δ-toxin has moderate cytolytic activity; and PSMβ peptides are non-cytolytic [31]. Although the exact mechanism of PSM toxicity against host cells is still unclear, the characteristic amyloid protofilaments assembled by stacking of amphipathic helices, which gives them surfactant-like characteristics, are proposed to result in the pathogenic activity in PSMα and PSMβ [32, 33].

**Table 1:** Major virulent mechanisms of Agr-related toxins

Toxin (gene)	Contribution of Agr expression on genes	Molecular character	Major virulent mechanism	References
PSMα (*PSMα*)	Activation (AgrA enhances *PSMα* promoter)	Amyloid protofilaments assembled by stacking of amphipathic helices	-Low concentration: stimulate leukocytes FPR2, leading to inflammatory response	[34]
			-High concentration:Cytolytic against leuckocytes, keratinocytes, and erythrocytes	[31, 35]
PSMβ (*PSMβ*)	Activation (AgrA enhances *PSMβ* promoter)		Unknown	
δ-toxin (*hld*)	Activation (*hld* encoded in RNAIII)		Mast cell degranulation	[36]
α-toxin (*hla*)	Activation (RNAIII initiate *hla* translation)	Pore-forming	-Pore forming cytotoxicity via ADAM10 binding in epithelial, endothelial, and immune cells	[37, 38]
			-Platelets aggregation	[39]
			-Inflammasome response in macrophages	[40]
protein A (*spa*)	Downregulation (RNAIII inhibit *spa* mRNA, RNAIII-*spa* mRNA degraded by RNAse III)	Five homologous Ig-binding domains	-Resist opsonization by Fc binding	[41, 42]
			-Activate TNFR1	[43]

PSMα plays a major role in the bacterial interaction with neutrophils. Neutrophils can directly respond to specific bacterial molecules, ‘‘pathogen-associated molecular patterns,’’ via Toll-like receptors (TLRs) [44] or certain G protein-coupled receptors (GPCRs), such as the formyl peptide receptor (FPR) family [45]. FPR recognize the formylated bacterial peptides and can activate host cells as well as elicit chemotactic migration. A formylated methionine is a hallmark of bacteria since only bacterial cells start protein biosynthesis with formylated methionine, whereas the human cells use an unmodified methionine for the initiation of translation [45]. At sub-cytolytic concentration, PSMα stimulate leukocytes via FPR2 and initiate pro-inflammatory responses, including neutrophil chemoattraction activation, and the release of interleukin (IL)-8 [34]. Once *S*. *aureus* is recognized by TLRs or GPCRs of immune cells, neutrophils migrate from vessels into tissues, phagocyte bacteria, and kill the bacteria. PSMα at high concentrations is able to lyse neutrophils after phagocytosis and induce a marked proinflammatory response while promoting bacterial survival [46, 47].

In contrast to the pro-inflammatory response in neutrophils, PSMα3 modulates monocyte-derived dendritic cells (moDC) to a tolerogenic phenotype in vitro. PSMα3 incubation with moDC led to impaired TLR2/4-induced maturation, decreased pro- and anti-inflammatory cytokine secretion, as well as reduced antigen uptake, and thus possibly increased the immune-tolerance toward the bacteria [48, 49].

Although very little is known about the role of PSMβ, it seems like PSMβ partly reverses the effect of PSMα on neutrophils and alleviate inflammation. This anti-inflammatory effect was seen in some reports, as measured by serum IL-6, neutrophil apoptosis in vitro, resulting in decreased host mortality in a mouse sepsis model [50] and a smaller dermonecrotic area in a subcutaneous injection model [31]. Meanwhile, δ-toxin is known to directly activate mast cells to degranulate [36].

In addition to the *psm* locus found in the core genome, some strains, particularly HA-MRSA, possess *psm-mec*. This is a mobile genetic element that contains both the *psm* and *mecA* genes. PSM-mec is responsible for antibiotic resistance and cytolytic capacity at the protein level. Interestingly, the *psm*-*mec* locus also encodes a regulatory RNA that inhibits the translation of the *agrA* gene [51]. The impact of this gene cassette on enhancing or inhibiting PSM expression is highly dependent on the specific strain, possibly due to the counteracting effects of the PSM-mec peptide and the RNA-controlled inhibitory effects of *psm-mec* [52, 53].

### Alpha-toxin

α-Toxin or α-hemolysin (hla) is a pore-forming toxin secreted as a soluble monomer [38]. α-Toxin assembles upon contact with the host disintegrin and metalloprotease 10 (ADAM10) to heptameric, β-barrel structure creating a cytolytic pore [37, 38]. Caveolin-1, the main component of the cell membranes, interacts with α-toxin and stabilizes the pore [54]. The *hla* mRNA normally forms a hairpin loop that prevents the ribosome from accessing its ribosome-binding site. RNAIII in the *agr* operon can bind to the *hla* mRNA, relieving the hairpin loop structure and allowing the ribosome to recognize the binding site for *hla* translation initiation [55]. Additional to Agr, *hla* expression levels can also be enhanced by the SarA regulatory systems as described above [24, 25, 56]. Although Agr appears to be the main regulator of *hla* expression, how other regulatory circuits contribute to *hla* expression in vivo remains unclear [57].

α-Toxin was initially named as α-hemolysin based on its property to lyse rabbit red blood cell, although later works revealed that human erythrocytes are devoid of ADAM10 and thus are insensitive to α-toxin [38, 58]. Rather, α-toxin intoxicates a wide range of human cell types via ADAM10 binding, including epithelial [38], endothelial [59], and immune cells, including T cells, monocytes, macrophages, and neutrophils [38, 60]. Additionally, recent works emphasize on α-toxin ability to cause the human platelets aggregation through ADAM10 interaction [39]. In vivo, α-toxin is an important virulence determinant contributing to skin necrosis in a subdermal injection model [61, 62], lethality in pneumonia [63, 64], and high bacterial burden in a brain abscess model [65]. α-Toxin contributes to the host lethal outcome in bloodstream infections [59] through disseminated thrombosis caused by platelets and neutrophil intoxication [39, 66]. The strong virulence of α-toxin depends on direct cell lysis as well as its ability to elicit host inflammatory responses. Intoxication with α-toxin induces inflammasome activation and result in IL-1β secretion and cell death in macrophages and monocytes [67]. The following inflammatory response leads to recruitment of various immune cells and reaction, leading to necrotic tissue injury [68].

Consistent with murine experiments, α-toxin-ADAM10 interaction poses a deteriorating effect on human. In patients with *OTULIN* (a linear deubiquitinase) haploinsufficiency, increased levels of linear ubiquitin caused the accumulation of caveolin-1 complexes in dermal fibroblasts, not in leukocytes [69]. Caveolin-1 accumulation enhanced the cytotoxicity of α-toxin and resulted in a life-threatening Staphylococcal disease of the skin and lungs [69]. The good news is, α-toxin-neutralizing antibodies could rescue the impaired cell-intrinsic immunity to α-toxin in these patients [69]. Meanwhile, human possesses an innate immune mechanism utilizing autophagy machinery to counteract α-toxin-induced toxicity. Upon recognition of bacterial and CpG DNA, host cells transfer ADAM10-bearing exosomes to the cell surface and expose decoy ADAM10 to trap α-toxin [70]. These studies suggest that genetic differences in α-toxin-ADAM10 signaling may produce phenotypic variation in human *S. aureus* infections.

Notably, although α-toxin contribution to host organ damage and lethality is evident, some studies report α-toxin as not being responsible for high bacterial load in the infection model. In a peritoneal infection model, α-toxin contributed to high mice lethality, but did not affect the remaining bacterial load in peritoneal cavity [71]. Additionally, in a corneal infection model, α-toxin contributed to high corneal damage, but did not affect bacterial load on the cornea [72]. α-Toxin deletion led to a small abscess formation in a subcutaneous injection model, but did not change the bacterial load [73]. Moreover, conditional knockout of ADAM10 in lung alveolar epithelium led to increased survival, but did not alter bacterial load in *S*.* aureus* pneumonia [64]. Thus, α-toxin-ADAM10 interaction is essential for progressive lethal disease, although it may not affect toxin-mediated control of the tissue bacterial load, depending on the conditions and model for in vivo assay (Table 1).

### Other Agr-regulated toxins

Transcriptome analysis revealed other toxins and enzymes positively regulated by the Agr system, such as serine proteases (SplA-F, SspA), cysteine proteases (ScpA, SspB), gamma-hemolysin (Hlg), and lipase (Geh) [26]. Among them, some proteases are known to contribute to virulence through proteolytic activity against specific targets. For instance, SspA targets the Fc region of immunoglobulins, degrading it and disrupting the effector function of antibodies [3]. This action leads to a partial loss of antigenic determinants of the antibody. Moreover, SspA damages tight junctions on keratinocytes, contributing to the development of atopic dermatitis. Another set of proteases, the six serine protease-like proteins (SplA-SplF), encoded in a single operon, trigger Th2 cytokines and induce the production of IgE antibodies in response to allergens [3]. This immune response is implicated in the development of various chronic airway diseases, including asthma and pneumonia.

### Toxins downregulated by Agr system

In contrast, RNAIII downregulates some surface proteins, including protein A (Spa) [26]. Protein A interferes with immune cells by (1) non-specifically binding to the Fc portion of IgG and escape phagocytes opsonization and (2) binding to the Fab region of IgM to serve as a B cell superantigen and cause B cell apoptosis [41, 42]. Protein A also activates tumor necrosis factor receptor (TNFR)1, a receptor for TNF-α on airway epithelium even without IgG, and elicits inflammatory response [43] (Table 1).

*Spa* gene expression is negatively controlled by Agr expression through two distinct mechanisms. First, RNAIII directly inhibits *spa* mRNA by RNA-RNA interactions, inhibiting access to the ribosome binding site [74]. Second, the complex formed between RNAIII and *spa* mRNA is also a substrate for RNAse III, thus RNAIII can also inhibit Protein A production by enhancing the degradation of *spa* mRNA [74]. Additionally, *spa* is repressed by SarA by binding and altering the mRNA turnover [75] (Table 1). Thus, Agr expression may sacrifice some virulence factors associated with surface proteins.

Other than Protein A, Agr has been described to generally downregulate adhesion factors, collectively referred to as microbial surface components recognizing adhesive matrix molecules (MSCRAMMs). However, more recent studies revealed that Agr does not regulate most MSCRAMMs in clinical strains [76].

### Interspecies quorum sensing between bacteria

In addition to *S*. *aureus*, other staphylococcal species also employ AIPs for Agr Quorum Sensing. Despite these species sharing a common AIP structure, there are variations in the amino acid sequences of several AIPs. Consequently, staphylococci with different AIP types engage in competitive interactions. They upregulate the expression of Agr in bacteria with the same AIP type, while concurrently downregulating the expression of Agr in other staphylococci with different AIP types [77].

### *S. aureus* necessitates functional Agr to colonize skin and cause Th2-driven skin inflammation in atopic dermatitis

**Fig. 2:**
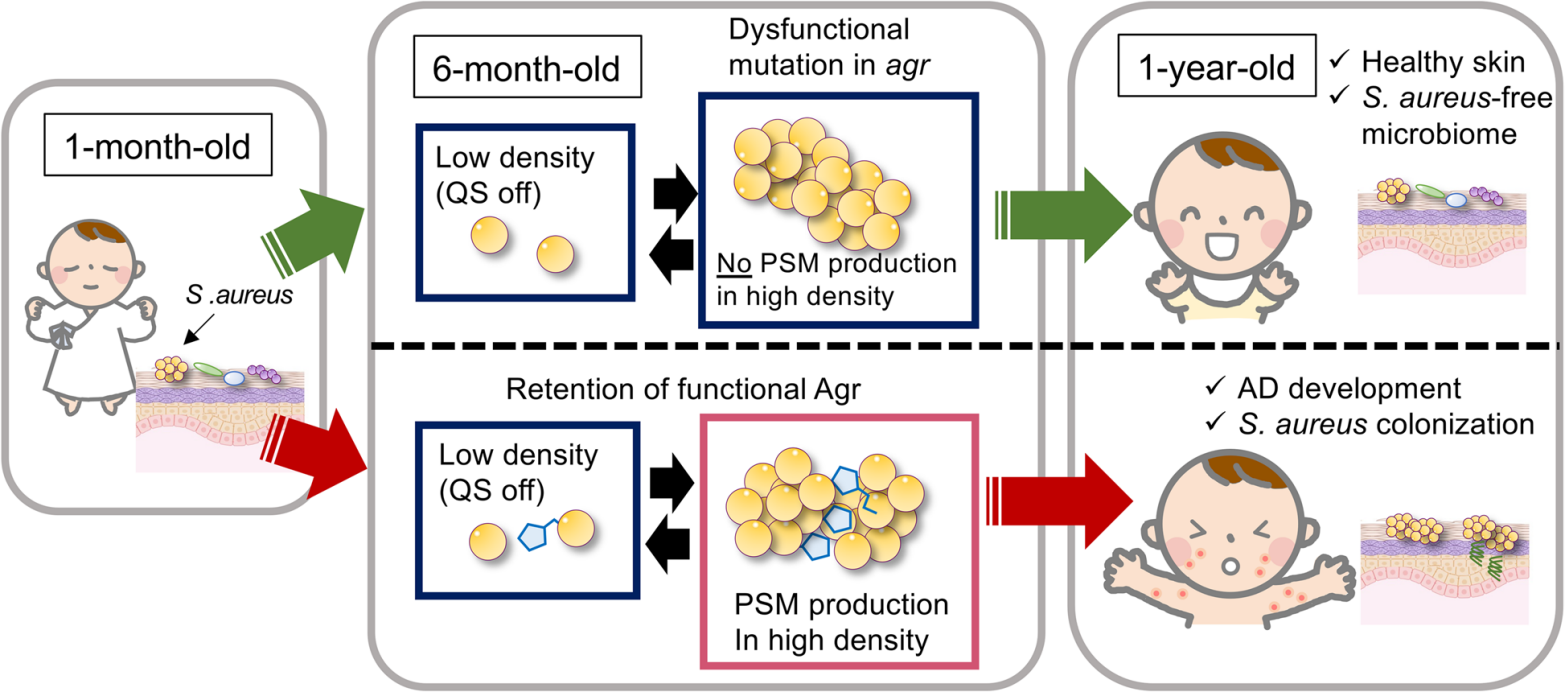
Retention of Agr in infant skin is related to atopic dermatitis. In a Japanese cohort study, *S*. *aureus* colonization at 1 month old did not affect the development of AD at 1 year old. However, possessing *S*. *aureus* on skin at 6 months old substantially increases the risk of developing AD at 1 year old. The whole-genome sequencing revealed that *S*. *aureus* on skin of the infants who did not develop AD by the age of one year acquired loss-of-function mutations in the *agr* locus between one and six months of age, whereas retention of a functional Agr is crucial for *S*. *aureus* to colonize the infants’ skin and cause AD. *AD* atopic dermatitis

In addition to its role as an opportunistic pathogen, *Staphylococcus aureus* can establish colonization on various human sites such as the skin, nares, and intestine. Notably, *S*. *aureus* skin colonization is strongly associated with atopic dermatitis (AD), a condition influenced by environmental factors, Th2 cell-skewed immunity, and deficiencies in the skin barrier [78]. The pathogenesis of AD is further complicated by alterations in the skin microbiome, known as dysbiosis. In fact, a significant percentage (30–100%) of AD patients are found to be colonized with *S*. *aureus*, in contrast to an approximate 20% prevalence in healthy control subjects [79, 80]. Moreover, the bacterial loads of *S. aureus* on the skin have been observed to correlate with the severity of AD [81, 82]. Despite these associations, the specific contribution of *S*. *aureus* to the pathogenesis of AD remained unclear until recent developments in research (Fig. 2).

**Fig. 3:**
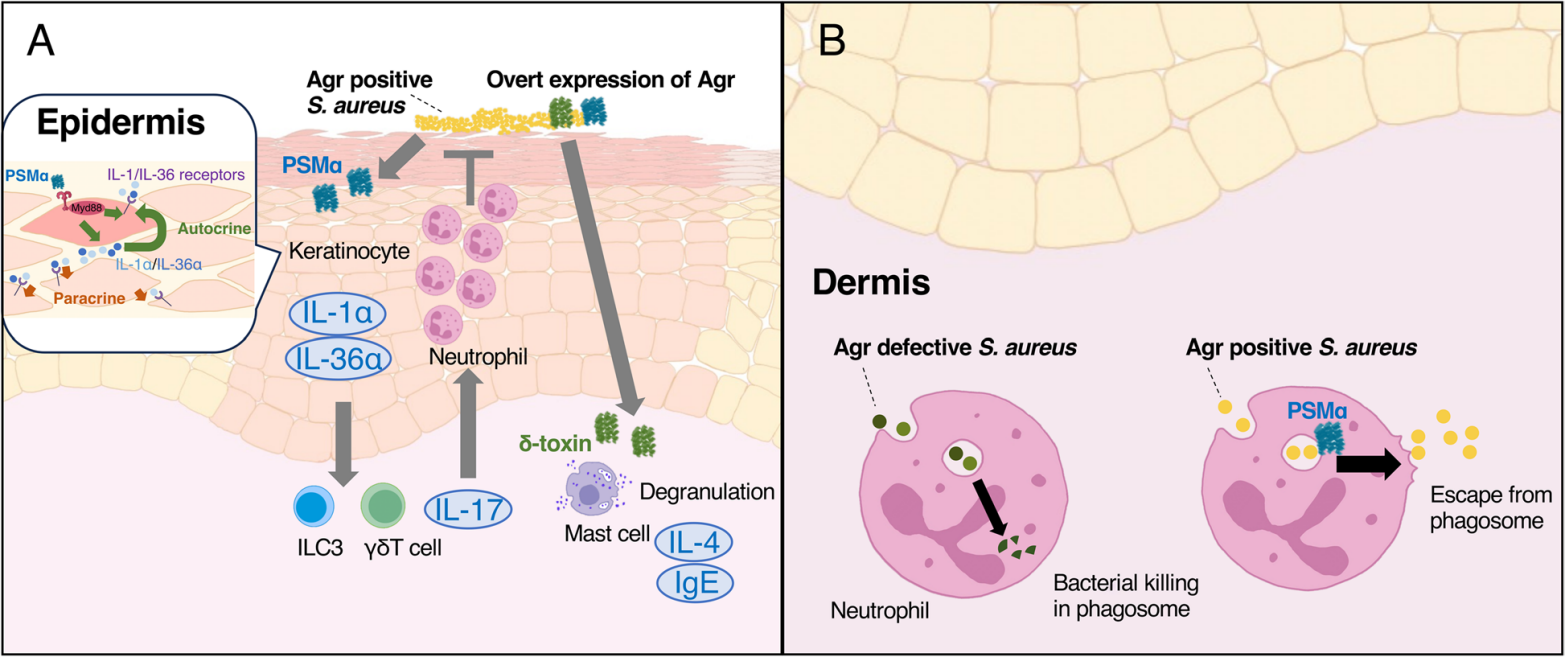
Agr-regulated toxins in atopic dermatitis. **A** Upon contact with *S*. *aureus*, keratinocytes detect PSMα, triggering the release of alarming signals, such as IL-1α and IL-36α. The receptors IL-1R and IL-36R become amplified during the inflammatory response in immunocompetent cells, leading to the induction of IL-17-producing γδ T cells and ILC3. IL-17 plays a crucial role in protective immunity against bacteria by promoting neutrophil recruitment. δ-toxin is a potent inducer of mast cell degranulation, contributing to the Th2-driven skin inflammation represented by IgE and IL-4 production. **B** Once bacteria reach the dermis, *S. aureus* utilizes PSMα to escape from phagosomes into the cytosol and limit both oxidative and non-oxidative pathogen killing after neutrophil engulfment. This leads to bacterial growth and consecutive inflammation in dermis. PSM: phenol-soluble modulin

The human skin microbiome is composed of bacteria, archaea, viruses, and fungi, and differ in communities at different body sites [83]. The infant skin microbiome is affected by various factors including delivery mode and neonatal skin barrier [84, 85], although the long-term consequences of these initial perturbations are not known. The early life microbiome undergoes frequent strain replacements over time [86]. During puberty, the sebaceous glands increase sebum production, and postpubescent skin favors lipophilic organisms [80, 87, 88]. The skin microbial communities in healthy adults remains stable, regardless of environmental perturbations [89]. However, dysbiosis associated with AD is characterized by decreased microbial diversity and an increase in *Staphylococcus* in general, especially *S*. *aureus* [79, 90–94]. Why and when *S*. *aureus* particularly colonizes AD-infected skin, especially on the lesional skin, remains unclear. One cohort study analyzed infants skin microbiome sequentially during 1 to 6 months after birth and found that approximately 45% of the infants were colonized with *S*. *aureus* in the cheek at 1 month, whether or not infants developed AD later in their life [13]. However, possessing *S*. *aureus* on skin at 6 months old substantially increases the risk of developing AD later in life [13]. Whole-genome sequencing of the bacterial genome showed that having a properly functioning Agr is crucial for *S*.* aureus* to colonize the infants’ skin and cause AD [13]. Additionally, a murine model of epicutaneous *S*. *aureus* colonization demonstrated that the Agr system plays a critical role in the epidermal colonization [13] (Fig. 3). Moreover, only Agr-positive strains could induce AD-like eczematous skin, as measured by skin disease score and histological analysis. Another study utilizing *Staphylococcus caprae* to inhibit the *S*. *aureus* Agr QS via AIP competition also exhibited that Agr-expressing *S*. *aureus* colonized on the skin of mice more efficiently than the Agr-suppressed strain [95]. Thus, functional *agr* seems to be necessary for *S*. *aureus* to colonize skin. This may explain why *S*. *aureus* on AD-infected skin can be a silent colonizer in a steady state, but also transform into a pathogenic phase with increase in number during an AD flare. Certain toxins reportedly playing key roles in the AD development are actually regulated by Agr, thus are only expressed when the bacteria reach high population densities. Specifically, *δ-*toxin and PSMα, which are regulated under the Agr system, are proven to elicit skin inflammation in AD. δ-toxin is a potent inducer of mast cell degranulation, contributing to the T helper 2 (Th2)-driven skin inflammation represented by IgE and IL-4 production in mice epicutaneous *S*. *aureus* infection models (Fig. 3, Table 1) [36]. Concomitant with mice data, *S*. *aureus* isolates recovered from patients with AD produced high levels of δ-toxin [36]. On the other hand, another study utilized AIPs derived from coagulase-negative staphylococci (CoNS), specifically *S*. *epidermidis*, to inhibit the Agr system of *S*. *aureus* and alleviated skin symptoms (M. R. [96]. This competitive interference highlights the intricate dynamics among different staphylococcal species and their impact on skin health.

Keratinocytes serve as the frontline defense against bacteria, actively sensing microbial presence beyond their role as a physical barrier. Upon contact with *S*. *aureus*, keratinocytes detect PSMα, triggering the release of alarming signals, such as IL-1α and IL-36α, with skin barrier disruption [35]. The receptors, IL-1R and IL-36R, become amplified during the inflammatory response, leading to the induction of IL-17-producing γδ T cells and Type 3 innate lymphoid cells [35] (Fig. 3, Table 1). IL-17 plays a crucial role in protective immunity against bacteria by promoting neutrophil recruitment, antimicrobial peptide production, and enhancing barrier function [97], among which neutrophils are essential for preventing *S*. *aureus* from invading the dermis [98]. Thus, PSMα-induced keratinocytes inflammatory response contributes to the protective immunity against *S*. *aureus*. Meanwhile, epicutaneous *S*. *aureus* colonization/infection enhances Th2-driven skin inflammation and skin barrier disruption, two important hallmarks of AD, via Agr-regulated toxins (Fig. 3).

Surprisingly, in neutrophil-deficient mice, *S*. *aureus* penetrates the epidermis with only mild Th2-driven skin inflammation and then grows in the dermis [98]. This epidermal penetration was dependent Agr and PSMα [98]. Once *S*. *aureus* reaches into subdermis, pathogen grow and expand more severely than neutrophil-sufficient mice, depending on *saeR/S* but no on Agr [98]*.* This discrepancy may at least partially explain why *S*. *aureus* requires Agr to cause SSTI in immunocompetent patients, but can infect immunocompromised hosts without the need for Agr.

### Agr system in skin and soft tissue infections

#### Clinical features of CA-MRSA

The most frequent disease manifestation associated with CA-MRSA is the skin and soft tissue infections (SSTI), accounting for at least 90% of CA-MRSA infections [99]. CA-MRSA SSTI are usually severe and often very painful. Up to 4% cases of CA-MRSA infection manifest as very potentially life-threatening skin infections, such as necrotizing fasciitis, whereas HA-MRSA rarely leads to such an invasive SSTI [100]. CA-MRSA strains also cause various infections such as osteomyelitis, pneumonia, sepsis, and urinary tract infections. The observation that CA-MRSA strains have the capacity to infect otherwise healthy people had indicated enhanced virulence. Many genetic and phenotypical analyses were attempted to establish the key differences of CA-MRSA and HA-MRSA, revealing Agr system as a significant key player in virulence.

### Molecular background of strong virulence in CA-MRSA

The predominant factors of enhanced virulence in CA-MRSA was initially believed to rely on the bacterial ability to evade phagocytes killing by Panton–Valentine leukocidin (PVL), a pore-forming toxin to kill immune cells [6, 101]. However, more recent research have questioned the importance of PVL as a major contributor to CA-MRSA virulence [63, 102–105] since an increasing number of CA-MRSA clones do not contain *lukSF* genes responsible for PVL [106] and *lukSF*-deficient clones are not less virulent than *lukSF*-containing CA-MRSA clones in animal experiments [107]. Rather, recent papers emphasize that the Agr system has a crucial role in CA-MRSA infection [62, 108, 109]. Many epidemiological studies report that dysfunctional Agr is higher among HA-MRSA (25–30%) versus CA-MRSA (up to 5%) [110–112]. Therefore, Agr plays a key role in CA-MRSA SSTI in vivo.

### Agr system in mice subcutaneous injection models

*S*. *aureus* can invade the host skin from minor scratches or wounds and may cause skin infections to become invasive [113]. However, most mouse experiments for *S*. *aureus* SSTI rely on subcutaneous bacterial injection to resemble cellulitis in human clinical settings [114]. SSTI in humans also occur without apparent skin barrier impairment, for example at hair follicles (folliculitis), deep (furuncles), or confluent abscesses (carbuncles) [115]. With subcutaneous bacterial injection model using Agr whole-knock out *S*. *aureus*, numerous studies established that Agr positive strains cause dramatically strong skin inflammatory responses, leading to abscess formation, skin necrosis, and ulcers with high bacterial load in the skin [108, 116, 117]. Hence, functional Agr seems to have an essential contribution on CA-MRSA virulence, in contrast to HA-MRSA. Among many toxins regulated by Agr, PSMα is proven to have a strong impact on bacterial burden and abscess formation in mice intradermal injection experiments [31, 62]. *S*. *aureus* relies on PSMα to escape from phagosomes into the cytosol and limit both oxidative and non-oxidative pathogen killing after neutrophil engulfment, to promote bacterial growth within the dermal layer (Fig. 3) [98]. Another study showed that *agr* whole-knock out strains show considerably less abscess formation and bacterial survival than PSM knockout strains, suggesting other Agr-regulated toxins than PSMα are responsible for *S*. *aureus* virulence in mice subdermal injection models [118].

In addition to invading host skin from minor scratches [113], *S*. *aureus* may be capable of actively disrupting the epithelial barrier function. α-Toxin activates ADAM10 on epithelial cells, thereby cleaving E-cadherin, which is one of the most important molecules in cell–cell adhesion [119]. Many studies report that α-toxin is an important virulence determinant in mice subcutaneous injection models, especially in eliciting skin necrosis [61, 119]. However, whether α-toxin is critical in disrupting intact skin barrier to invade and cause subcutaneous infection remains to be elucidated. A recent study has emphasized PSMα function in *S*. *aureus* penetrating epidermis to the dermis in neutrophil-deficient mice [98].

### *S. aureus* Agr system in bacteremia

*S. aureus* is one of the most common causes of bloodstream infections worldwide [120]. The all-cause mortality rate from *S*. *aureus* sepsis in high-income countries has been reported to be up to 20–50% [120–123] and the recurrence rate reported to be 5–10% [124]. Entry of *S*. *aureus* into the bloodstream occurs mostly via colonization of intravenous catheters or dissemination from skin and soft tissue infections [125, 126]. *S*. *aureus* bacteremia can lead to secondary infectious foci in almost any tissue, resulting in a diverse range of infections, including infective endocarditis, tissue abscesses, meningitis, osteomyelitis, and septic arthritis. The bacterial capacity to infiltrate and disseminate to a broad range of second host tissue infections is the distinct characteristics of *S*. *aureus* infection. The extensive array of toxins supporting bacterial virulence are collectively termed by their function as, adhesins (attachment to host cells), invasins (penetration into host cells), and evasin (evasion of the host’s immune response), of which some of these effector molecules are at least partially regulated by the Agr system [127, 128]. Additionally, S.* aureus* bacteremia can lead to endothelial damage, platelet aggregation, and overt inflammatory responses, resulting in life-threatening disseminated intravascular coagulation (DIC). The DIC microthrombi further damage the endothelium and block blood flow, resulting in oxygen depletion in organs, as well as depleting available clotting factors and paradoxically causing hemorrhages [129]. In this pathophysiology, endothelial damage and platelet activation are related to α-toxin under Agr regulation.

It is therefore easy to understand that the Agr system is required for systemic infection, as numerous studies have shown in different animal models of infection. However, in the real-world settings, 3–82% of cases of *S*. *aureus* bacteremia are caused by strains lacking detectable Agr activity [130–132]. Moreover, dysfunctional Agr is reported to be an independent risk factor for MRSA bacteremia-attributed mortality [8]. Notably, *S*. *aureus* sepsis and systemic infections mostly happen in patients in the hospital settings, not in healthy individuals. Some epidemiological studies revealed that the healthcare environment selects for loss of Agr function and carriage of *agr*-defective strains is strongly associated with a hospital stay or prior use of antibiotics [111, 133–135]. Some studies using clinical isolates revealed that *agr* mutants do not transfer between patients [111] and the dysfunctional mutation of Agr occurs newly in every individual infection rather than in a population-wide process [135]. However, we still do not know why the lack of Agr can be beneficial for *S*. *aureus* to cause bacteremia and other diseases in immunocompromised patients. There seems to be limitations with animal models analyzing each virulence factor independently to approach the dramatic pathogenesis of infections in the host. Nevertheless, taking various data into consideration, it appears that *S*. *aureus* benefits from both expressing and not expressing Agr, depending on the specific phase of the infection and the location of the pathogen within the host [136]. In the bloodstream, apolipoprotein B in serum sequesters AIP, blocking Agr activity, and any produced toxins will be quickly diluted. Therefore, Agr-regulated toxins do not contribute to sepsis severity [137]. Meanwhile in organs, maintaining a functional Agr system is useful for surviving inside phagocytes and establishing a niche in the host [136]. When *S*. *aureus* clumps or reside inside host cells, high bacterial density allow them to activate Agr and produce toxins. At the later stage of local infection, bacteria benefit from not expressing Agr to have strong adhesion to organs and avoid eliciting immune responses [136]. We will separately review the benefit of expressing and not expressing Agr in systemic infections in the following section.

### α-Toxin and PSMα enable S. aureus to survive intracellularly in phagocytes

**Fig. 4:**
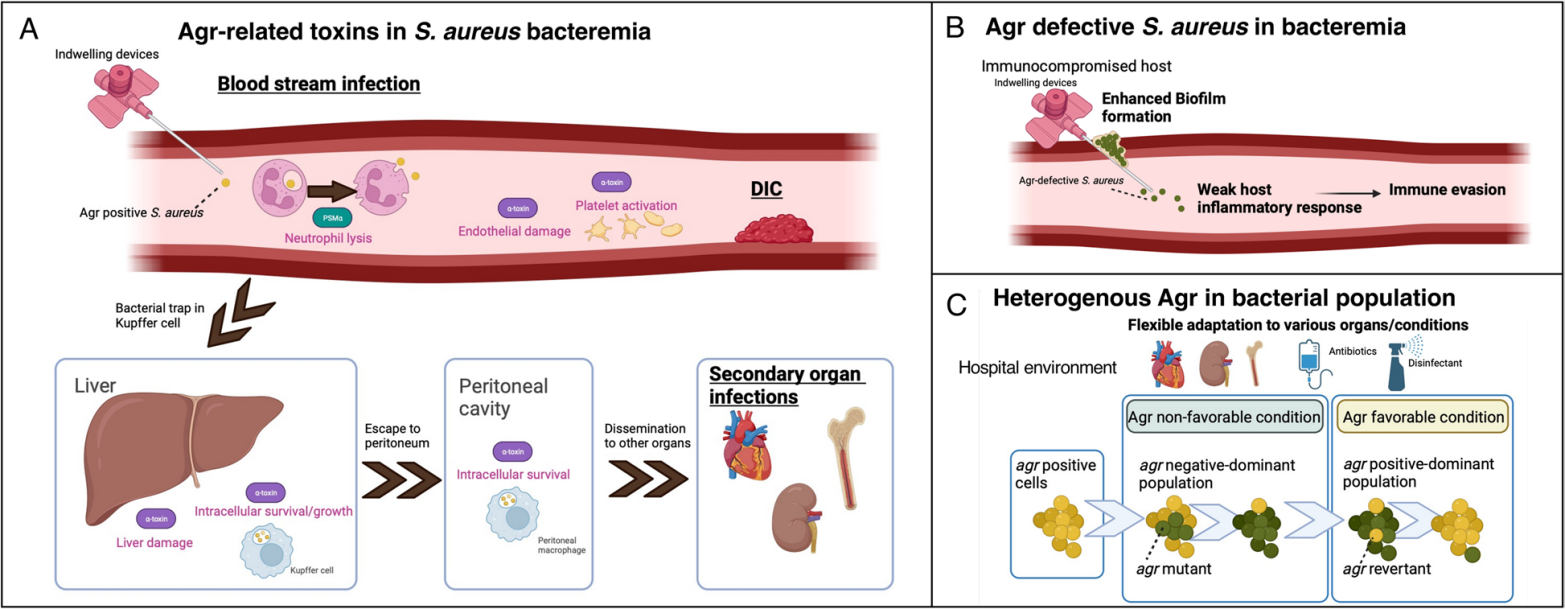
Agr system in systemic infection. **A** In the bloodstream infection, *S*. *aureus* PSMα can lyse neutrophils after phagocytosis, resulting in re-entry of the pathogen into the bloodstream. Additionally, *S. aureus* α-toxin can lead to endothelial damage, platelet aggregation, and overt inflammatory responses, resulting in life-threatening disseminated intravascular coagulation (DIC). In mice *S*. *aureus* sepsis experiments, bacteria are trapped in Kupffer cells, but survive and multiply within cells, escape to the peritoneum and become trapped in peritoneal macrophage, and eventually disseminate to other organs. α-toxin is involved in the liver damage and intracellular survival in this pathogenesis. **B** Agr defective *S*. *aureus* reportedly produces dense biofilm and escapes from immune attack by not eliciting strong inflammatory response. **C** *S*. *aureus* population is not always homogeneous in Agr activity but can produce an *agr* mutant or revertant within them to coordinately survive in various conditions in hospitals. *PSM* phenol-soluble modulin

Recent research revealed the role of liver Kupffer cells and peritoneal macrophages as infectious reservoirs in* S*. *aureus* bacteremia [138, 139]. In mice *S*. *aureus* sepsis experiments, bacteria are trapped in Kupffer cells, but survive and multiply within cells, escape to the peritoneum and become trapped in peritoneal macrophage, and eventually disseminate to other organs (Fig. 4) [138, 139]. This intracellular survival is a critical mechanism that determines the development of subsequent *S*. *aureus* bacteremia and the establishment of infection in other organs [140]. In the initial phase of Kupffer cells engulfing *S*. *aureus*, platelets rapidly bind to the Kupffer cells, preventing escape of the pathogen [141]. At the later phase of Kupffer cell-*S*. *aureus* interaction, platelet aggregation caused by α-toxin induces microthrombi and subsequent liver damage [66]. Also in human macrophages, α-toxin is a key effector molecule essential for *S*. *aureus* intracellular survival [40]*.* Besides, *S*. *aureus* PSMα can lyse neutrophils within 2 to 4 h after phagocytosis, resulting in re-entry of the pathogen into the bloodstream (Fig. 4) [46, 47]. Bacteria may repeat this cycle by being taken up by nearby healthy neutrophils or, alternatively, disseminate to other sites causing secondary infection foci [142]. Thus, Agr plays a critical role in *S*. *aureus* sepsis by facilitating bacterial survival inside phagocytes and even using phagocytes as carriers for dissemination. Additionally, α-toxin also activates platelets and endothelial cells in sepsis, ultimately leading to DIC (Fig. 4).

### Benefit of not expressing Agr in bacteremia

Agr defective clinical isolates seem to arise in a low-cell-density state particularly in cases with endocarditis, osteomyelitis, and bacteremia [143–145]. Despite the loss of toxin production, there is evidence that *agr*-defective strains are considerably more likely to cause persistent infection than *agr*-competent strains, resulting in an increased rate of secondary infections and mortality [130, 131, 146–149]. The deregulation of Agr seems to somehow confer an advantage in certain host niches and many studies attempt to reveal how these mutations enable *S*. *aureus* to infect the host. Some plausible explanations for the prevalence of *agr*-defective *S*. *aureus* strains causing bacteremia are (1) Agr-regulated toxins may not be necessary for infecting immune-compromised hosts, (2) *agr*-defective *S*. *aureus* have enhanced ability to form a biofilm, and (3) *S*. *aureus* benefit from other toxins than Agr-regulated toxins.

### Agr-defective S. aureus escape host neutrophil attack

Notably, Agr expression enables bacteria to successfully escape from phagocytes killing; however, it supposedly leads to a strong immune response, which eventually reduces bacterial survival (Fig. 4). In vitro studies reported that Agr-positive strains trigger a strong pro-inflammatory response in neutrophils, including IL-8 and TNF-α expression, compared with that of Agr negative strains [46]. However, in mice sepsis models, *S*. *aureus* lineage with an attenuated form of PSMα elicited increased bacterial burden on bloodstream, with diminished cytolytic and chemotactic activity toward human neutrophils [27, 28]. Thus, Agr defective strains may evade recognition and subsequent elimination by host neutrophils, thereby successfully disseminating during blood infection [27, 28].

### Biofilm formation in agr mutants

The contamination of indwelling medical devices is another route of infection that occurs frequently in the hospital setting [150]. *S*. *aureus* can form biofilms, which is a multicellular bacteria embedded in an extracellular matrix to protect them from phagocyte attacks and killing.* S*. *aureus* can form biofilms on various types of abiotic surfaces, such as indwelling medical devices, as well as tissue surfaces, such as heart valves in the case of endocarditis [150]. Biofilm formation starts from bacterial attachment to a surface, production of the extracellular matrix, and the disassembly of the biofilm to disseminate to other sites [150]. Many studies attempted to reveal molecular mechanisms of biofilm formation, although this dynamic process seems to be orchestrated by a complex network and the whole process remains unclear [150]. Among many regulatory systems involved in biofilm formation, Agr reportedly affects biofilm formation either negatively or positively depending on the formation step [150].

Many studies showed that the Agr system is necessary for efficient *S*. *aureus* dissemination from a biofilm infection and subsequent spreading into neighboring tissues, mainly depending on PSMs [151–153]. Another study reported *S*. *aureus* Agr activation, particularly the PSM production, is also a key component in biofilm structuring [152]. An in vivo study using a murine orthopedic implant biofilm infection model showed that macrophage phagocytosis and cytotoxicity decrease with the biofilm, which is partially dependent on Agr [154]. However, Agr-dysfunctional strains formed dense and enlarged biofilms [155]. Moreover, clinically isolated *S*. *aureus* gained *agr* mutation during infection to cause device-associated infection and increased biofilm formation [135]. Some possible explanations of contraindications in reports are (1) Agr may function differently on biofilm production and dispersal [156]; (2) the *S*. *aureus* population in a biofilm is not always homogeneous in Agr activity, but can produce an *agr* mutant or revertant within them to coordinately form biofilm [118, 135]; and (3) many environmental conditions such as pH, glucose level, and attached surface affect biofilm formation, thus making culture-based experiments difficult to reproduce the same environment as in vivo [150].

To overcome this, recent work used the subcutaneous catheter infection model in which catheter pieces were coated with bacteria and inserted under the dorsal skin of mice for 6 days before bacterial loads in the biofilm were analyzed (Fig. 4) [118]. In this model, Agr-dysfunctional cells formed larger biofilms than that of Agr-positive cells and had increased resistance toward neutrophil attacks in immunocompetent mice [118]. Additionally, in a subcutaneous catheter-associated and prosthetic joint-associated infection model, sub-inhibitory concentrations of antibiotics increased the incidence of *agr* mutation, leading to a considerable increase in bacteria and the bacterial load [157]. In these animal models relevant to clinical situations in hospitals, Agr-defective strains seem to succeed in creating dense biofilm and eventually cause bacteria compared to Agr-positive strains. However, this thesis remains to be further assessed with other infection models, since human serum affects the *S*. *aureus* transcriptome and behavior [158], thus biofilm production may differ in the subcutaneous space from blood vessels. A suitable model of catheter-associated biofilm infection, such as a study inserting an indwelling device in blood vessels [159], can be used for further study.

### Uninhibited protein A production in Agr-defective *S. aureus*

As mentioned in the previous section, Agr activation suppresses some toxin expression. Among them, protein A functions as an essential virulence factor in *S*. *aureus* platelet aggregation, forming an abscess, and eliciting inflammation. Protein A activates platelet aggregation via its binding to von Willebrand factor [160] and is a virulence determinant in endovascular infection in a rabbit model of endocarditis [161]. Additionally, protein A mutants are unable to form abscesses, although the mechanism remain unknown [162]. As abscess formation shield bacteria from host immune cells by a surrounding pseudo-capsule and enable bacteria to replicate inside, lack of abscess formation leads to quick bacterial elimination [163]. Moreover, in airway epithelium, protein A stimulates TNFR1, and contributes to pathogenesis of *Staphylococcus*-related pneumonia [43]. Thus, expressing protein A instead of Agr-regulated toxins may contribute to virulence in various settings. The prophylactic or therapeutic use of anti-protein A succeeded in improving the survival rate and diminishing bacterial load in mice sepsis and peritoneal infection model, suggesting that protein A is critical in these infection models [164].

In another study analyzing a clinically isolated *agr* defective strain, there were multiple genetic changes in virulence factors (such as the *S*. *aureus* ESAT6-like secretion system) other than the *agr* system, which resulted in increased virulence in a murine model of bloodstream infection. Thus, there was a partial compensation for the absence of conventional *agr*-mediated virulence with another virulence factor [133]. Although the functional Agr is likely crucial in infection, its importance may be substantially diminished in some situations, and Agr-defective strains may cause mortality through other virulence factors [123, 165–167].

## Conclusions

Despite extensive research on *S*. *aureus*, there is still a notable absence of effective treatments or preventive measures for bacterial infections caused by this organism, apart from antibiotic therapy. The challenge stems from the complex expression or suppression of numerous virulence factors by this bacterium, influenced by diverse environmental conditions such as host immunity, organ specificity, and antibiotic utilization, resulting in the wide array of intricate phenotypes. Another challenge in understanding the pathogenicity of *S*. *aureus* lies in the reliance on mouse infection models for many research efforts, despite mice not being the natural host for *S*. *aureus*. Additionally, certain secreted factors exhibit toxicity in a strain-specific manner [e.g., CHIPS (a chemotaxis inhibitory protein of staphylococci) and PVL], further complicating the understanding of the pathogenic mechanisms [168, 169].

This review aimed to enhance our understanding of *S*. *aureus* pathogenesis by focusing on Agr, as a critical gene regulation system impacting the bacterial phenotype. Altogether, Agr-regulated toxins are essential in causing SSTI in immune-competent patients. However, *agr*-defective strains seem to cause sepsis and secondary infections in immunocompromised hosts, of which bacteria utilize multiple toxins to move between organs and live in a specific niche. Notably, while some infection types may select for entirely *agr*-functional or *agr*-defective populations, other infections yield mixed populations (Fig. 4) [170]. Moreover, *agr* mutations occur while bacteria are infecting patients, to cope with selective pressures [135]. In vitro, the *agr* revertant rise within a population possibly because bacteria cannot acquire essential nutrients in a population completely devoid of Agr-controlled secreted degradative enzymes [118]. Thus, instead of conducting experiments that directly compare Agr-expressing and non-expressing strains, a more realistic approach may involve a model where a fixed percentage of both Agr-expressing and non-expressing strains are mixed in a population. This dynamic system, where the percentage changes over time, could provide insights into more realistic phenomena and the dynamic interactions between these strains and hosts. The pathophysiology of *S*. *aureus* infection is substantially influenced by phenotypic changes resulting from factors beyond Agr. Future studies are expected to give the comprehensive understanding of *S. aureus* overall profile in various settings.

## Data Availability

Not applicable.

## Abbreviations

AD: Atopic dermatitis
Agr: Accessory gene regulator
AIP: Auto-inducing peptide
CA-MRSA: Community-associated MRSA
DIC: Disseminated intravascular coagulation
FPR: Formyl peptide receptor
GPCR: G protein coupled receptor
HA-MRSA: Hospital-acquired MRSA
*hla*: α-Hemolysin
*hld*: Delta-hemolysin
HPK: Histidine protein kinase
IL: Interleukin
moDC: Monocyte-derived dendritic cell
MRSA: Methicillin-resistant *Staphylococcus* *aureus*
PSM: Phenol-soluble modulin
QS: Quorum sensing
SSTI: Skin and soft tissue infection
TLR: Toll-like receptor
